# Cardiovascular sexual dimorphism in a diet-induced type 2 diabetes rodent model, the Nile rat (*Arvicanthis niloticus*)

**DOI:** 10.1371/journal.pone.0208987

**Published:** 2018-12-27

**Authors:** Jillian Schneider, Sharee Kuny, Donna Beker, Yves Sauvé, Hélène Lemieux

**Affiliations:** 1 Department of Physiology, University of Alberta, Edmonton, AB, Canada; 2 Department of Ophthalmology and Visual Sciences, University of Alberta, Edmonton, AB, Canada; 3 Department of Pediatrics, University of Alberta, Edmonton, AB, Canada; 4 Faculty Saint-Jean, Women and Children's Health Research Institute, Dept. of Medicine, University of Alberta, Edmonton, AB, Canada; Scuola Superiore Sant'Anna, ITALY

## Abstract

**Background:**

The Nile rat **(*Arvicanthis niloticus*)** is an emerging laboratory model of type 2 diabetes. When fed standard rodent chow, the majority of males progress from hyperinsulinemia by 2 months to hyperglycemia by 6 months, while most females remain at the hyperinsulinemia-only stage (prediabetic) from 2 months onward. Since diabetic cardiomyopathy is the major cause of type-2 diabetes mellitus (T2DM)-related mortality, we examined whether sexual dimorphism might entail cardiac functional changes. Our ultimate goal was to isolate the effect of diet as a modifiable lifestyle factor.

**Materials and methods:**

Nile rats were fed either standard rodent chow (Chow group) or a high-fiber diet previously established to prevent type 2 diabetes (Fiber group). Cardiac function was determined with echocardiography at 12 months of age. To isolate the effect of diet alone, only the small subset of animals resistant to both hyperinsulinemia and hyperglycemia were included in this study.

**Results:**

In males, Chow (compared to Fiber) was associated with elevated heart rate and mitral E/A velocity ratio, and with lower e’-wave velocity, isovolumetric relaxation time, and ejection time. Of note, these clinically atypical types of diastolic dysfunction occurred independently of body weight. In contrast, females did not exhibit changes in cardiovascular function between diets.

**Conclusions:**

The higher prevalence of T2DM in males correlates with their susceptibility to develop subtle diastolic cardiac dysfunction when fed a Western style diet (throughout most of their lifespan) despite no systemic evidence of metabolic syndrome, let alone T2DM.

## Introduction

Type 2 diabetes (T2DM) is facing a worldwide epidemic with more than 422 million affected, and a persistent increasing trend [1]. The evolution of the most deadly T2DM complication, cardiomyopathy, still eludes us [2]. A major hurdle to research efforts has been that animal models typically entail either gene modifications, chemical treatment (ex. streptozotocin or alloxan injections), or acute dietary induction (ex. high sugar/fat feeding) [3]. These models fail to mimic the prolonged and sustained chronic complex physiological events that, compounded with age, insidiously culminate to detectable T2DM in human.

The Nile rat, *Arvicanthis niloticus*, is a promising laboratory model typified by an interplay of genetic and dietary factors that together lead to slow and progressive T2DM development with similar complications as observed in human [4–6]. In contrast to most rodents, which are more resistant than human to diabetes under *ad libitum* access to Westernized diets [7], this species is not [6]. Nile rats fed standard laboratory rodent diet develop T2DM over the course of a year. Such slow progression overlaps with aging, a well-established risk and compounding factor in T2DM and cardiovascular diseases [8]. Diabetic cardiovascular complications include accelerated atherosclerosis, systolic and diastolic dysfunction, left ventricular hypertrophy, atrial fibrosis and fibrillation, heart failure, and increased post myocardial infarction fatality [2]. When T2DM is superimposed with aging, these complications develop on average 14.6 years faster [9] and with greater severity [10].

The strong sexual dimorphism with regard to T2DM development (affecting predominantly males) offers the added opportunity of addressing sex-related differences in disease progression. In human, the prevalence of diagnosed cases in adults is similar between men and females (9.4% versus 8.9%, respectively), although undiagnosed cases are more frequent in males (3.8%) than females (2.3%), particularly so for hyperinsulinemia (40.2% in males and 27.8% in females) [11]. Furthermore, disease-related mortality rate in males (54.2%) exceeds that of females (45.8%) [11]. Male Nile rats fed standard laboratory rodent chow spontaneously exhibit pathological markers of metabolic syndrome and protracted T2DM [5, 6, 12]. Most males undergo insulin resistance and hyperinsulinemia by 2 months and hyperglycemia by 6 months. In contrast, the majority of females remain prediabetic from 2 months onward [6]. Feeding *ad libitum* high-fiber low-fat diet effectively prevents hyperglycemia and associated complications in both male and female Nile rats, throughout their 18–24 months lifespan [6].

To validate the Nile rat as a model of T2DM-induced cardiovascular complications, we firstly sought to document baseline cardiac function in a clinically analogous manner. Taking into account the well-documented diet and sexual dimorphism in this species, baseline echocardiography parameters were defined in relation to sex and nutritional statuses. Whereas most male Nile rats are diabetic at 12 months [6], our study relied on the less than 20% minority that had normal blood insulin and glucose levels in order to isolate the direct contribution of diet (a modifiable lifestyle factor) on male versus female cardiac function. Of note, animals selected for this study had similar body weight within each sex groups, which excluded the potential contribution of obesity, a well-established risk factor for T2DM and cardiovascular diseases [13]. Finally, these conditions precluded that any preventative effects of dietary fibers on cardiovascular dysfunctions might be indirectly mediated *via* changes in body weight [14].

## Materials and methods

### Animals

Male Nile grass rats (*Arvicanthis niloticus*) were obtained from a colony at the animal facility of the University of Alberta, which was started in 2006 with breeders generously provided by Dr. Laura Smale from her own colony at Michigan State University (supported by a grant from the National Institute for Mental Health). Animals were maintained on a 14:10 h light-dark cycle, at 21±2°C room temperature and ~40% relative humidity. After weaning at 21–23 days, animals were divided into two dietary groups: 1) Fiber group, fed Mazuri Chinchilla (5M01, Purina Mills, LLC, St. Louis, MO, USA; 4.0% fat, 15.3% fibers, 21.6% protein); 2) Chow group fed Prolab (RMH 2000, 5P06, LabDiet, Nutrition Intl., Richmond, IN, USA; 9.6% fat, 3.2% fibers, 19.9% protein). With the only exception of fasted blood glucose measures done at 12 hours fasting, animals had *ad libitum* access to food and water. The University of Alberta Institutional Animal Care and Use Committee approved this study (protocol 328). In addition, all experiments were undertaken in accordance with and the NIH (USA) guidelines regarding the care and use of animals for experimental procedures.

### Echocardiography

Echocardiography was performed at 12 months by adapting a procedure originally developed for rats [15]; see protocols.io (identifier: dx.doi.org/10.17504/protocols.io.u83ezyn). In brief, following mild anesthesia with isoflurane delivered through a nosecone (VEVO Compact Anesthesia system), animals were sedated with 3% isoflurane and maintained under anesthesia using 2% isoflurane, throughout the procedure (~30 min) while monitoring ECG, heart rate (S1 File), and respiratory rate. Body temperature was kept at 37°C (measured rectally) using a warmed platform (VisualSonics). Cardiac parameters were imaged using the Vevo 770 High-Resolution In-Vivo Micro-Imaging System (Fujifilm VisualSonics Inc.) and a 17.5 MHz Scanhead probe to achieve high-frequency ultrasound imaging. The upper anterior chest (superior to the zyphoid process) was shaved and fine hairs were removed using Nair depilatory cream. Warmed ultrasound gel was liberally applied immediately prior to imaging. Ventricular dimensions were measured using M-mode transthoracic echocardiography at the papillary muscles level, during a minimum of 3 continuous cardiac cycles (S1 Fig). The following three M-mode-wall measurements were recorded at both end-diastole and end-systole: left ventricular internal diameters (LVID), intraventricular septal wall thickness (IVS), and left ventricular posterior wall thickness (LVPW). Thereafter, the ejection fraction (EF) and fractional shortening (FS) were obtained with the following formulas:

EF(%)=[(LVEDV–LVESV)/LVEDV]X100

$$FS\;\left(\%\right)=\left[\left(LVID_{d}‑LVID_{s}\right)/LVESV\right]\;X\;100$$

Where LVEDV and LVESV represent the left ventricular end diastolic volume and end systolic volume, respectively.

Left ventricular mass was also calculated from M-mode images according to the uncorrected cube function formula:

$$LV\;mass=1.053\;X\;\left[{\left(LVIDd+LVPWd+IVSd\right)}^{3}–LVIDd^{3}\right]$$

Where 1.053 is the specific gravity of the myocardium.

Measures of aortic ejection time (ET) from these waveforms allowed determining the myocardial performance index (TEI index) as followed:

TEIindex=(IVRT+IVCT)/ET.

Pulse wave Doppler of the mitral E-wave and A-wave velocities were taken from the four chambers view (S2 Fig), and used to assess diastolic function parameters including isovolumetric relaxation time (IVRT) and isovolumetric contraction time (IVCT), E/A, deceleration time of E-wave. Finally, septal valve annular velocities, e’ and a’ were determined by tissue Doppler imaging from the apical four chamber view (S3 Fig).

### Metabolic phenotype

Just prior to euthanasia (intra-peritoneal injection of a lethal dose, 480 mg kg^-1^, of pentobarbital sodium; Euthanyl Bimeda-MTC Animal Health Inc., Cambridge, ON, Canada), fasting blood glucose (FBG; after 16–18 hours fasting) levels were assessed as and plasma samples collected for insulin measures, as described previously [16]

FBG > 5.0 mmol l^-1^ reflected hyperglycemia [6]. Following euthanasia, animals were weighted and measured.

### Data analysis

Statistical analyses were performed with SigmaPlot 14 (Systat Sofware Inc., San Jose, CA, USA). Graphics were produced using GraphPad Prism 7 (GraphPad Software, Inc., La Jolla California). The echocardiography data were analyzed using a two-way anova with diet and sex as the two factors. Criteria of normality and homogeneity of variance for ANOVA were tested for each variable using Kolmogorov-Smirnov (Lilliefor’s correction) and Brow-Forsythe tests, respectively. Data are presented as median (min-max) without transformation, where N is the number of animals. Significance was set at p<0.05, using two-tails comparisons.

## Results

### Body weight and FBG level

**Fig 1:**
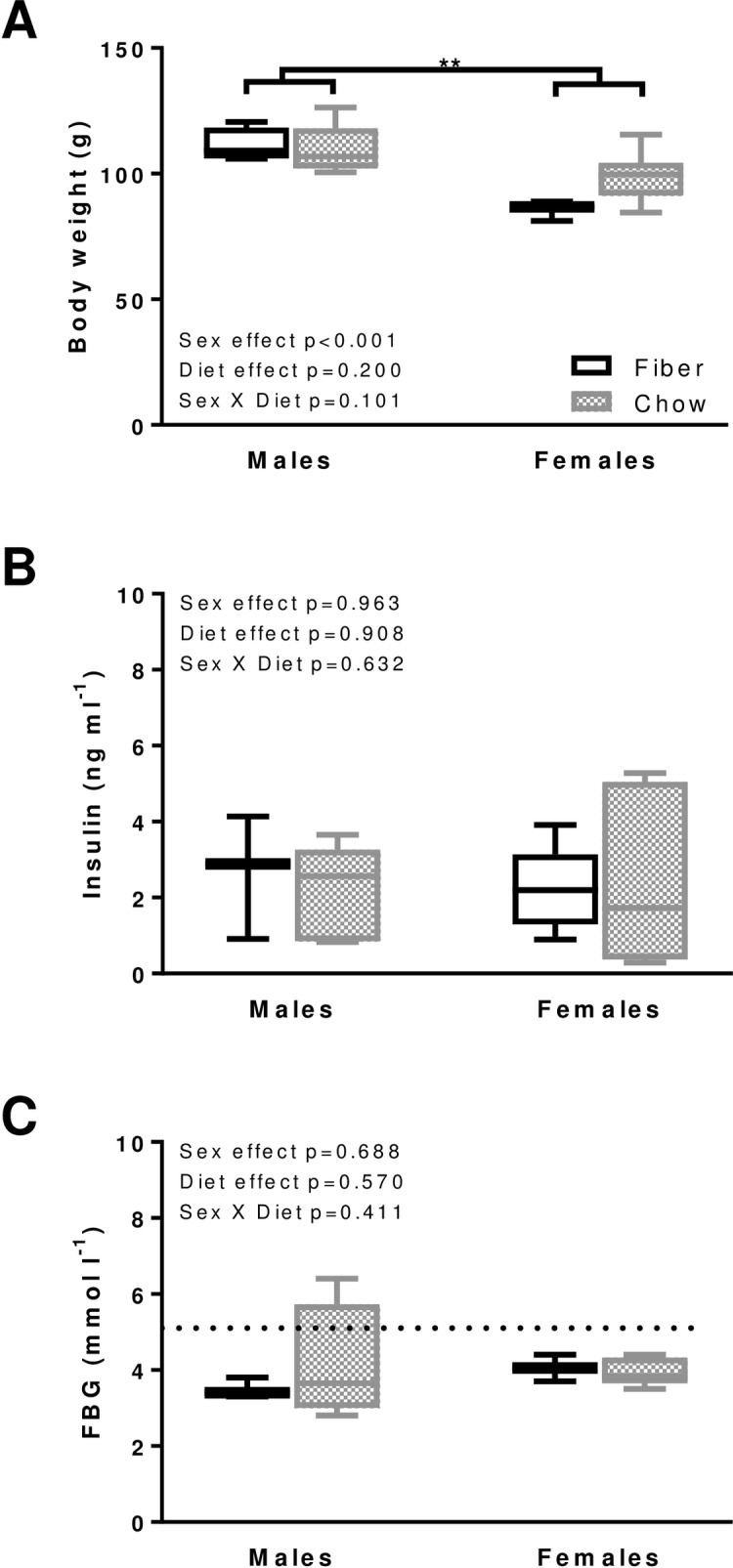
Metabolic phenotype in the males and females from the Fiber (black boxes) and Chow (grey boxes) groups. Body weight (A), fasting blood insulin (B) and glucose (FBG, C) were measured. Boxes indicate the minimum, 25^th^ percentile, median, 75^th^ percentile, and maximum. Significant differences between dietary groups or sexes are indicated with ** for p<0.01. In males, N equals 4 and 5 for body weight and 3 and 4 for insulin and FBG in the Fiber and Chow group, respectively. In females, N equals 3 and 6 for body weight, insulin and FBG in the Fiber and Chow group, respectively. Kruskal-Wallis results are indicated on each panel; data are from the same animals for which echocardiography was performed, with the exception of Fiber females for the insulin data only, and Chow females which represent a distinct group of age-matched animals.

In order to study cardiac function independently of the effect of obesity [17], hyperinsulinemia [18] and high-glycemic load [19], we selected animals within each sex group that had similar body weight as well as normal insulin and FBG levels (Fig 1). Only one male in the Chow group had borderline high FBG value (6.4 mmol l^-1^, compared with 5.6 mmol l^-1^ threshold).

### Cardiac morphometry

**Fig 2:**
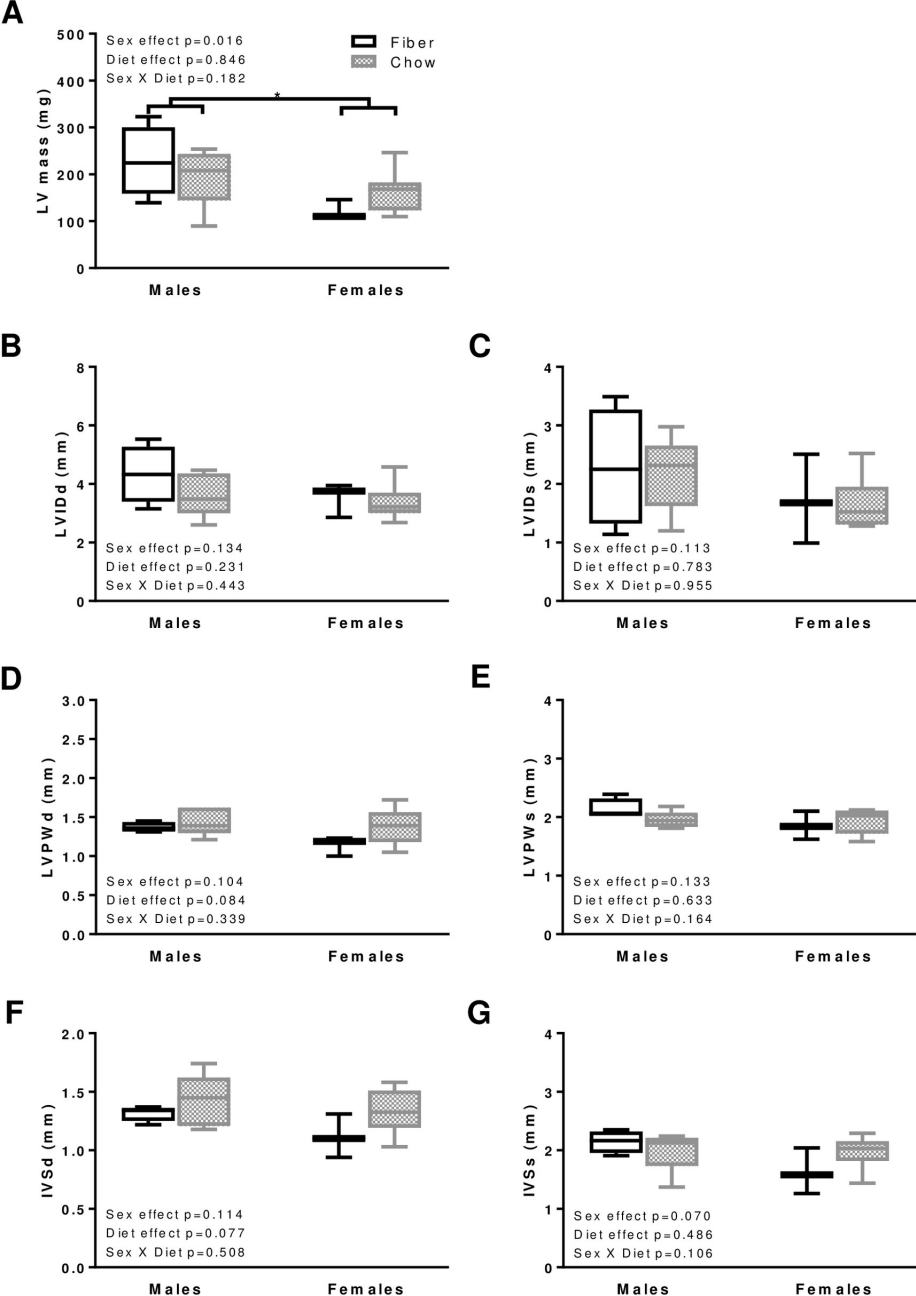
Morphology in males and females from the Fiber (black boxes) and Chow (grey boxes) groups. The following parameters were measured: left ventricular (LV) mass (A), left ventricular internal diameter (LVID, B and C), left ventricular posterior wall thickness (LVPW, D and E), interventricular septum wall thickness (IVS, F and G) at end-diastole (d, left panels B, D, F) and end-systole (s, right panels C, E, G). Boxes indicate the minimum, 25^th^ percentile, median, 75^th^ percentile, and maximum. BPM indicates beat per minute. In males, N equals 4 and 5 for Fiber and Chow groups, respectively. In females N equals 3 and 8 in Fiber and Chow groups, respectively. Significant differences between dietary groups or sexes are indicated with * for p<0.05. Kruskal-Wallis results are indicated on each panel.

While unaffected by diet, the left ventricular mass was inferior in lower weight animals (females) compared to males (p = 0.016; Fig 2A). There were no differences in the left ventricular internal diameter (Fig 2B and 2C), the left ventricular posterior wall thickness (Fig 2D and 2E), or the interventricular septum wall thickness (Fig 2F and 2G). Overall, the morphometric results rule out any effects of diet or sex on concentric left ventricular hypertrophy or asymmetrical septal hypertrophy [20].

### Systolic function

**Fig 3:**
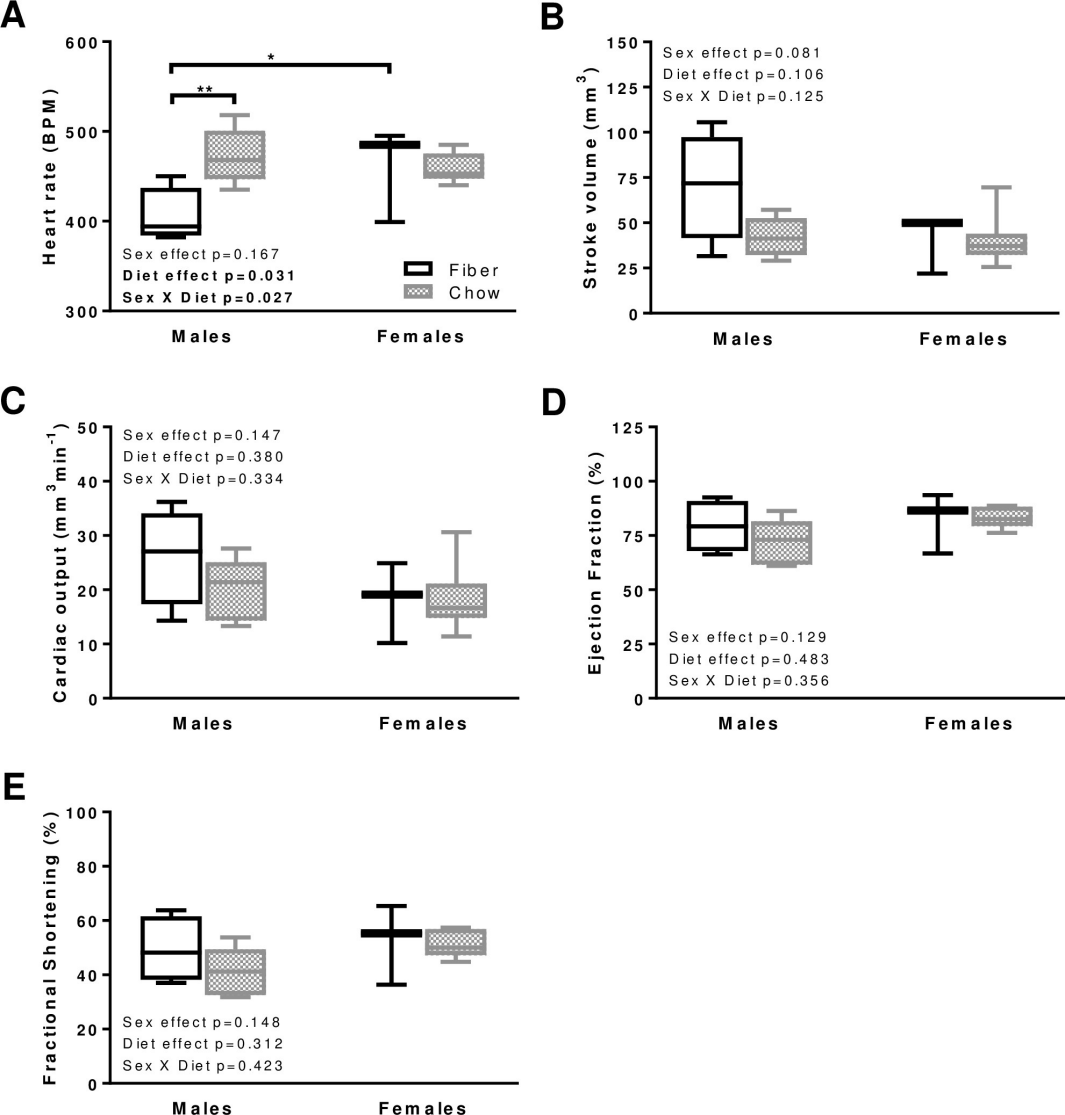
Systolic function in males and females from the Fiber (black boxes) and Chow (grey boxes) groups. The following indicators were measured: heart rate (A), stroke volume (B), cardiac output (C), ejection fraction (D), fractional shortening (E). Boxes indicate the minimum, 25^th^ percentile, median, 75^th^ percentile, and maximum. In males, N equals 4 and 5 in the Fiber and Chow group, respectively. In females N equals 3 and 8 in the Fiber and Chow group, respectively. Significant differences between dietary groups or sexes are indicated with ** for p<0.01 and * for p<0.05. Kruskal-Wallis results are indicated on each panel.

Left ventricular systolic performance is an indicator of heart disease severity and is therefore used as a predictor of morbidity and mortality [20]. Despite similarities in body weight and FBG between dietary groups, heart rate was faster in males from the Chow compared to Fiber group (p = 0.004; Fig 3A). For the Fiber group, males had lower heart rates than females (p = 0.028; Fig 3A). This sexual dimorphism was not observed in the Chow group (Fig 3A). The main indicators of systolic function were not affected by either diet or sex; these included stroke volume (Fig 3B), cardiac output (Fig 3C), ejection fraction (Fig 3D), and fractional shortening (Fig 3E). Only one parameter was affected by diet, and in females only: the systolic velocity at the mitral annulus (s’) was slower with the Fiber compared to the Chow group (S1 Table; p = 0.011). Of note, the Nile rat's chest has a unique barrel-like shape that represents a challenge when attempting to obtain accurate measurements related to the left ventricle. As such, systolic velocity findings should be interpreted with caution.

### Diastolic function

**Fig 4:**
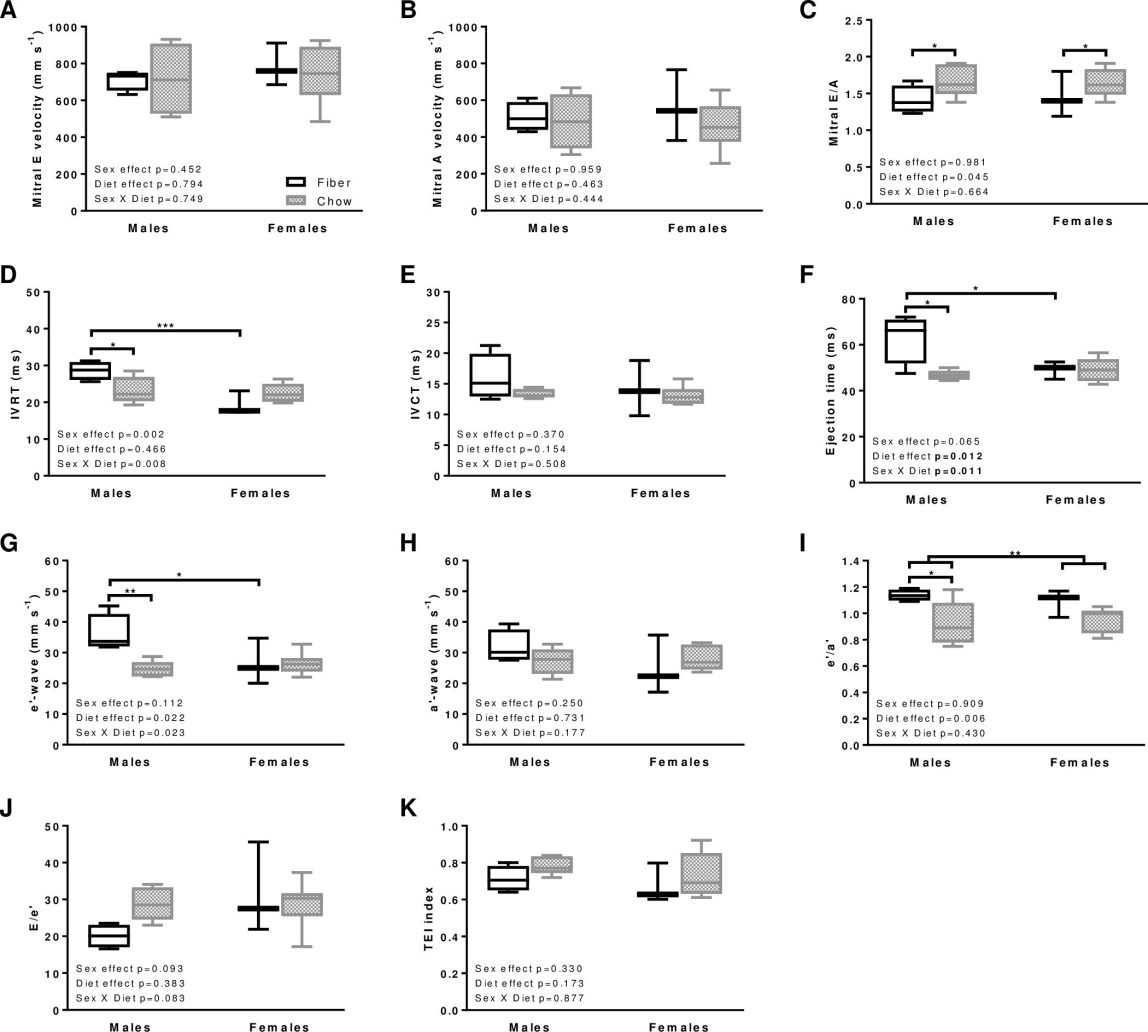
Diastolic function in males and females from the Fiber (black boxes) and Chow (grey boxes) groups. The figure includes mitral E velocity (A), mitral A velocity (B), E/A ratio (C), isovolumetric relaxation time (IVRT, D), isovolumetric contraction time (IVCT; E), ejection time (F), tissue Doppler e’-wave velocity (G), a’-wave velocity (H), ratio e’/a’ (I), ratio E/e’ (J), and myocardial performance index (TEI index, K). Boxes indicate the minimum, 25^th^ percentile, median, 75^th^ percentile, and maximum. In males, N equals 4 and 5 in the Fiber and Chow group, respectively. In females N equals 3 and 7–8 in the Fiber and Chow group, respectively. Significant differences between dietary groups or sexes are indicated with *** for p<0.001, ** for p<0.01 and* for p<0.05. Kruskal-Wallis results are indicated on each panel.

Left ventricular diastolic dysfunction is an important factor to consider in heart failure [20, 21]. In this context, it is a strong generic predictor of mortality, independently of systolic functional status [22]. Mitral inflow and tissue Doppler images were used to investigate potential diastolic dysfunction (Fig 4). Pulse waves Doppler of the mitral E-wave (Fig 4A) and A-wave (Fig 4B) velocities did not vary between dietary groups. In contrast, diet exerted an effect on the E/A ratio with higher values in both sexes from the Chow compared to Fiber group (Fig 4C; p = 0.045).

Diastolic function was further assessed by measuring IVRT, IVCT, E-wave deceleration time and ejection time. In males, IVRT was of shorter duration in the Chow compared with the Fiber group (Fig 4D; p = 0.016). Within the Chow group, IVRT was also reduced when compared with females (p<0.001; Fig 4D). These results point to restrictive filling in Chow males. In contrast, IVCT (Fig 4E) and deceleration time (S1 Table) did not vary between sexes (p = 0.370 and p = 0.657, respectively) or dietary groups (p = 0.154 and p = 0.731, respectively). Ejection time in males was shorter in the Fiber compared to the Chow group (p = 0.001; Fig 4F), which could be explained, at least in part, by the inverse correlation between heart rate and ejection time [23, 24].

Tissue Doppler imaging from the apical 4-chamber view was used to measure mitral tissue septal annulus velocities, e’ and a’, which reflect tissue movement at the septal valve leaflet. In males, e’-wave velocity was slower in the Chow compared to the Fiber group (Fig 4G; p = 0.007), while a’-wave values were unchanged (Fig 4H), yielding to reduced e’/a’ ratios (Fig 4I). In females, diet did not exert an effect on parameters e’ or a’ (Fig 4I). Other related parameters where also unaffected by diet or sex: the E/e’ ratio (Fig 4J) and the myocardial performance index that incorporates both systolic and diastolic time intervals to provide a global assessment of ventricular function (Fig 4K).

## Discussion

Type 2 diabetes is a well-established risk factor for cardiovascular diseases [25–27], with males being more affected than females [11]. We used the Nile rat model of slowly progressing T2DM to investigate the effect of diet and sex on cardiac function. Our findings indicate that diet-induced diastolic changes occur in animals showing resistance to metabolic syndrome and T2DM, independently of body weight (males had similar fasting insulin and blood glucose levels as well as body weight in both dietary groups studied here). Our focus on males and females resilient to pre-diabetes and diabetes adds further support for the possibility that cardiac functional changes might occur insidiously at an early (preclinical) stage in T2DM. The changes observed consist of elevations in heart rate and mitral E/A ratio as well as reductions in e’-wave velocity, isovolumetric relaxation time, and ejection time. These results support diastolic dysfunctions, albeit, in an atypical fashion when compared to more common clinical symptoms of diabetic cardiomyopathy in human. Despite having selected animals without any detectable systemic defects, males nevertheless exhibited diet-related cardiac dysfunction while females did not. Our findings support the occurrence of an underlying sex-based susceptibility to dietary intake over a prolonged period (almost throughout lifespan) and provide further insights into the well-established sex-differences in T2DM-associated cardiovascular dysfunction [28].

The diet-induced cardiovascular changes occurring in males solely are in line with previous findings of left ventricular diastolic dysfunction as an early complication in human T2DM (reviewed in [28, 29]). These dysfunctions arose in prediabetic patients [30], in subjects with impaired glucose tolerance but without diabetes [31, 32], and in young patients with diabetes, but without atherosclerosis, obesity, or hypertension, [33, 34]. In prediabetic patients, compared to controls, a strong difference in coronary blood flow reserve, which predominantly builds during diastole, was measured. This was accompanied with more modest changes in baseline and peak systolic and diastolic blood pressure, and no changes in E/e’, A/E, IVRT, and Tei index [30]. In patients with impaired glucose tolerance (as opposed to normoglycemic individuals), a decrease in E/A ratio [31, 32] and increase in IVRT [31] has been documented. IVRT, a marker of myocardial relaxation, refers to the interval between the closure of the aortic valve, and the opening of the mitral valve, which indicates filling onset and the first phase of diastolic relaxation [35]. A prolonged IVRT reflects poor myocardial relaxation and often manifests itself with aging [36]. Interestingly, in contrast with human, cardiovascular dysfunction was absent in studies using fructose fed rat [37, 38], despite development of hyperglycemia after only 6 weeks of high fructose feeding. These observations point to aging as a T2DM compounding factor, which does make a slow progression model like Nile rats even more relevant to study early cardiac dysfunction, prior to hyperinsulinemia and hyperglycemia onset.

In males, not females, long-term feeding of a Western-style diet is accompanied with elevated early to late ventricular filling velocity ratios and, higher E/A ratios, which together support an abnormal pattern of mitral inflow [39, 40]. Whereas E/A ratios also vary with heart rate, their inverse correlations [41–45] do not explain our results since heart rate and E/A ratio were both higher in Chow-fed compared to Fiber-fed males. Furthermore, we detected differences in early peak velocities (e’) and early to late diastolic peak velocity ratios (e’/a’) in Chow compared to Fiber males. Lower e’/a’ ratios are indicative of abnormal mitral inflow [39, 40], with optimal sensitivity when taken at the septal rather than lateral annulus such as was the case in the present study [46]. The ratios of e’/a’ were previously shown to correlate with left atrial volume indexes (indicator of left ventricular diastolic function) [47], independently of potential confounders such as age, sex, body weight index, hypertension, hyperinsulinemia and diabetes [46]. Finally, another indication of diastolic dysfunction in males fed a Western-style diet was the lower e’- versus a’- wave velocities, yielding e’/a’ ratio below 1, compared with over 1 in males fed a high fiber diet. Observations of e’-wave being slower than a’-wave do support impaired left ventricular relaxation and is now widely used as a diagnostic tool of diastolic dysfunction [39, 40, 48, 49]. Septal e’ is a more optimal diagnostic tool compared to lateral e’ [30]. Long-term feeding on a Western-style also affects IVRT, although, in an opposite fashion (reduction) compared to that in human (prolongation) [31]. Whereas our results suggest restricted left ventricular filling, one must take into account the contribution of dietary-induced changes on heart rate itself. The higher heart rate associated with Western diet in males implies that each phase is accelerated and therefore could explain our observations of reduced IVRT and ejection time. Our study clearly points out signs of diastolic dysfunction such as changes in (1) the relationship between early and late filling (E- and A-wave), (2) the ratio of mitral valve annular velocities (e’/a’), (3) the elevated filling pressure (E/e’), and (4) the time needed to fill the ventricle after relaxation (IVRT). By definition, diastolic dysfunction implies that the cardiac ejection fraction (systole) is preserved, while cardiac relaxation (diastole) is disrupted.

Diet-associated increases in heart rates in males are particularly instructive when considering that these did not occur in females. Higher fiber feeding is better aligned with the overall metabolic scaling in animals [50], with higher heart rates in animals with smaller size (females) compared to larger size (males). Such relationship does not hold when males are fed a Western-style diet: the heart rate is comparable between males and females, despite males having larger body weights. Previous studies using a mouse model of diet-induced T2DM (high fat diet for 24 weeks) revealed that animals of both sexes underwent similar increases in heart rate [51]. Even if we were to hypothesize that long term feeding of a Western-style diet in our study would have been associated with obesity in both sexes, the mechanisms explaining increased heart rates are sex-specific: a reduced cardiac vagal tone in females and a compensatory mechanism to preserve basal blood pressure while adrenergic contractility is reduced in the males [51]. The higher heart rate specific to males fed a Western-style diet (none of which developed obesity) might reflect compensatory mechanisms and/or increased stress levels. High-fat meals have been associated with heightened cardiovascular reactivity to stress [52] and increased sympathetic nervous system activity [53]. Further studies will be required to determine whether chow-fed males had reduced cardiac efficiency due to increased reliance on fatty acid oxidation for their energy production [54]. Fatty acid utilization increases the needs to rely on oxygen for oxidative phosphorylation [55]. In line with this, previous studies have shown that changes in capacity to metabolize fatty acids do take place at an early stage during T2DM [38] and heart failure [56].

Sexual dimorphism in electrocardiography parameters have been documented extensively in adults [57–63], children [64], and several animal models [65–67]. These include, among others, differences in LV mass [58, 68–73], left ventricular filling pressure [59–63], systolic function [59–63], tracing amplitude and duration [64], repolarization dynamics [74], and T wave generation [75]. These differences cannot be explained solely by variation in body size or in left ventricular mass [58]. Our findings in males *versus* females fed a high-fiber diet (IVRT, ejection time, e’-wave, heart rate) add to the body of literature on sexual dimorphism. Of note, these sex differences are absent when animals are fed Chow. This is in line with findings from individuals with type 1 diabetes, where differences in cardiovascular function between males and females are minimized [76]. Here again, an important and neglected parameter in animal studies is aging, as male cardiovascular health is more susceptible to aging [77]. Male Nile rats in our study showed signs of sexual dimorphism attributable alone to aging. In Nile rats, sexual dimorphism in T2DM susceptibility might reflect a contribution of sex hormones as male symptoms are exacerbated during puberty-associated testosterone production, but abate in females when estrus evolves around 7–8 weeks of age [4]. Based on previous findings [78–80], Subramaniam et al. [4] suggested that estrogen might exert a protective effect against diabetes, partly through microbiota modification. Interestingly, both sex hormones and microbiota have been suggested to play a role in cardiovascular function [81].

### Study limitations

Performing echocardiography on Nile rats represents technical challenges. Their barrel-shaped chest displaces the heart from the location anticipated in mice and rats, leading to more variability in tissue Doppler parameters, or even (in some cases) to the an inability to measure key parameters such as mitral inflow pattern, pulmonary venous flow pattern, or systolic velocity at the annulus. An intermediate sized echocardiography probe (between commercially dedicated rat and mouse sizes) would have been ideal, but such specialized device was not available. Also, Nile rats present the advantage (and limitation) of being a complex model of T2DM, involving diet and genetic parameters. In this model, Chow-fed animals segregate into susceptible (>80%) or resistant (<20%) subgroups when fed identical Western-style diets. However, it was previously reported that the few animals surviving up to 24 months on such diabetogenic diet all eventually develop metabolic syndrome or even diabetes [5, 82–84]. The limited availability of such disease resistant animals (at least up to 18 months in this study) restricted our findings to a low animal N number. Confirmation of a likely bimodal distribution in disease progression and of the respective disease time courses of these two populations will require further studies with much larger sample sizes. These larger-scale studies would also allow appreciating how age-related changes in cardiac function might segregate with sex. Due to their feral nature, Nile rats are not compliant with assessments of blood pressure, as the required restraints increase cortisol levels leading to artifactually elevated pressure in both groups. We cannot therefore control for the potential contribution of hypertension [12] on cardiac function. Notwithstanding, exclusion of animals presenting with hyperinsulinemia or hyperglycemia might have selected for no or mild hypertension phenotype. Finally, animals were not fasted prior to echocardiographic assessment, adding potential variability from the contribution of the current metabolic condition toward the parameters measured.

## Concluding remarks

The present data provide evidence of that long-term feeding with a Western-style diet leads to diastolic dysfunction in males, but not females. As heart failure with preserved ejection fraction is becoming more clinically relevant [22, 85], it is important to gather information relevant to left ventricular diastolic function in animal models. Our data show that despite resilience to metabolic syndrome and T2DM, protracted changes in cardiac function can occur under sustained Western-style diet. The current ubiquitous use of acute (as opposed to chronic) animal models of diabetes precludes studying and documenting these clinically important events [38]. Our observations point to the critical contribution of aging in pre-diabetic as a compounding factor for the insidious development of preclinical cardiovascular phenotypes. Of note, the majority of clinical diagnoses of T2DM are in seniors who had no previous history. Therefore, our findings further confirm our current clinical inability to identify, let alone understand, the chronic events (prior to hyperinsulinemia and hyperglycemia) that will eventually lead to cardiovascular complications.

## Supporting information

S1 Table Echocardiography data from males (M) and females (F) fed either Fiber or Chow diets. (DOCX)

S1 Fig Representative Mmode images in the parasternal short axis view and taken at the mid-papillary level. The image was from the heart of (A) a female in the Chow group, (B-C) two males in the Chow group.
(DOCX)

S2 Fig The representative mitral valve images shows pulse wave velocities of the E and A waves, taken in a modified apical 4-chamber view. The images are from the heart of (A) a female in the Fiber group, (B) a female in the Chow group, and (C) a male in the Chow group.
(DOCX)

S3 Fig The representative image of the tissue doppler shows the tissue velocities of the E' and A' taken in a modified apical 4-chamber view at the position of the septal leaflet on the mitral valve annulus. The images are from the heart of (A) a male in the Fiber group and (B) a female in the Fiber group.
(DOCX)

S1 File Multimedia files. The representative B-mode videos taken in the parasternal long axis view for a total of 7 animals.
A) Male in the Fiber group.
B) Male in the Fiber group.
C) Male in the Chow group.
D) Male in the Chow group.
E) Female in the Chow group.
F) Female in the Chow group.
G) Female in the Chow group.
(ZIP)
