# Supplementary material for: Cardiovascular sexual dimorphism in a diet-induced type 2 diabetes rodent model, the Nile rat (Arvicanthis niloticus)
Source: PLoS One. 2018 Dec 27;13(12):e0208987. doi: 10.1371/journal.pone.0208987 (PMC6307866; doi:10.1371/journal.pone.0208987)
Supplement: S1 Table — (DOCX) [file pone.0208987.s001.docx]

S1 Table.

| **Sex** | **Diet** | **Body weight** | **Fasting blood glucose** | **Insulin level** | **IVSd** | **LVIDd** | **LVPWd** | **IVSs** | **LVIDs** | **LVPWs** | **Stroke Volume** | **Cardiac Output** | **EF** | **FS** | **LV Mass** | **Heart Rate** | **Mitral E velocity** | **Mitral A velocity** | **E-wave Deceleration Time** | **IVRT** | **IVCT** | **Ejection Time** | **TEI Index** | **Doppler e'-wave velocity** | **Doppler a'-wave velocity** | **s'** |
| --- | --- | --- | --- | --- | --- | --- | --- | --- | --- | --- | --- | --- | --- | --- | --- | --- | --- | --- | --- | --- | --- | --- | --- | --- | --- | --- |
|  |  | **(g)** | **mmol l^-1^** | **ng ml^-1^** | **mm** | **mm** | **mm** | **mm** | **mm** | **mm** | **mm^3^** | **mm^3^ min^-1^** | **%** | **%** | **(mg)** | **BPM** | **mm s^-1^** | **mm s^-1^** | **ms** | **ms** | **ms** | **ms** |  | **mm s^-1^** | **mm s^-1^** | **mm s^-1^** |
| M | Fiber | 120.5 | 3.4 |  | 1.37 | 4.15 | 1.39 | 2.24 | 1.88 | 2.10 | 71.4 | 27.6 | 85.8 | 54.7 | 218.29 | 382 | 745.94 | 525.13 | 20.83 | 31.25 | 21.25 | 72.00 | 0.73 | 31.8 | 28.4 | 35.4 |
| M | Fiber | 106.5 |  | 2.890 | 1.34 | 3.15 | 1.33 | 2.35 | 1.14 | 2.39 | 31.6 | 14.3 | 92.6 | 63.8 | 139.4 | 450 | 716.47 | 428.35 | 18.25 | 25.62 | 12.50 | 47.50 | 0.80 | 32.7 | 27.5 | 30.1 |
| M | Fiber | 111.9 | 3.8 | 4.140 | 1.34 | 4.50 | 1.31 | 1.91 | 2.62 | 2.03 | 72.3 | 26.5 | 72.7 | 41.7 | 230.8 | 390 | 632.41 | 474.12 | 17.50 | 30.00 | 15.83 | 67.50 | 0.68 | 34.6 | 31.9 | 16.0 |
| M | Fiber | 105.9 | 3.3 | 0.914 | 1.22 | 5.53 | 1.45 | 2.09 | 3.49 | 2.02 | 105.6 | 36.2 | 66.3 | 37.0 | 323.3 | 398 | 751.62 | 611.42 | 18.75 | 27.50 | 14.37 | 65.00 | 0.64 | 45.24 | 39.4 | 26.3 |
| M | Chow | 109.9 | 3.6 | 2.559 | 1.24 | 4.24 | 1.38 | 2.24 | 2.33 | 1.97 | 57.2 | 27.6 | 76.7 | 45.0 | 208.0 | 483 | 930.90 | 667.69 | 21.06 | 19.26 | 13.11 | 45.21 | 0.72 | 28.7 | 29.2 | 38.0 |
| M | Chow | 126.3 | 2.8 | 2.871 | 1.45 | 4.47 | 1.39 | 2.13 | 2.98 | 1.86 | 47.9 | 22.5 | 62.2 | 33.3 | 253.8 | 468 | 838.45 | 526.69 | 13.65 | 22.22 | 13.89 | 46.67 | 0.77 | 24.6 | 32.8 | 35.0 |
| M | Chow | 106.8 | 6.4 | 0.881 | 1.50 | 3.40 | 1.62 | 2.10 | 2.32 | 1.94 | 35.4 | 15.4 | 61.0 | 31.8 | 199.4 | 435 | 583.65 | 439.79 | 16.67 | 28.52 | 12.96 | 50.00 | 0.83 | 22.4 | 27.8 | 24.0 |
| M | Chow | 100.5 | 3.7 | 0.825 | 1.18 | 2.60 | 1.21 | 1.37 | 1.20 | 1.81 | 29.0 | 13.3 | 86.3 | 53.8 | 89.9 | 459 | 510.16 | 304.63 | 14.44 | 25.19 | 14.44 | 47.22 | 0.84 | 22.2 | 25.0 | 25.0 |
| M | Chow | 103.7 |  | 3.651 | 1.74 | 3.48 | 1.62 | 2.18 | 2.05 | 2.18 | 41.3 | 21.4 | 73.1 | 41.2 | 233.1 | 518 | 715.13 | 620.32 | 11.31 | 21.48 | 12.59 | 44.44 | 0.77 | 25.1 | 21.3 | 33.0 |
| F | Fiber | 81.2 | 3.7 | 0.894* | 1.31 | 2.86 | 1.19 | 2.04 | 0.99 | 1.84 | 21.9 | 10.2 | 93.6 | 65.4 | 110.0 | 485 | 759.5 | 543.1 | 8.1 | 17.7 | 13.8 | 50.0 | 0.628 | 34.7 | 35.7 | 20.3 |
| F | Fiber | 88.9 | 4.05 | 3.913* | 0.94 | 3.95 | 1.00 | 1.26 | 2.51 | 1.62 | 49.9 | 19.1 | 66.7 | 36.4 | 109.8 | 399 | 685.7 | 381.4 | 17.5 | 23.1 | 18.8 | 52.5 | 0.798 | 25.0 | 22.3 | 26.0 |
| F | Fiber | 86.8 | 4.4 | 2.363* | 1.10 | 3.76 | 1.23 | 1.58 | 1.68 | 2.10 | 50.8 | 24.9 | 86.5 | 55.3 | 146.1 | 495 | 911.1 | 766.2 | 26.9 | 17.3 | 9.8 | 45.0 | 0.601 | 20.0 | 17.1 | 17.5 |
| F | Fiber |  |  | 1.700* |  |  |  |  |  |  |  |  |  |  |  |  |  |  |  |  |  |  |  |  |  |  |
| F | Fiber |  |  | 2.199* |  |  |  |  |  |  |  |  |  |  |  |  |  |  |  |  |  |  |  |  |  |  |
| F | Chow | 84.6* | 3.8* | 0.464* | 1.03 | 3.01 | 1.05 | 1.44 | 1.32 | 1.58 | 32.0 | 14.8 | 87.8 | 56.3 | 109.7 | 464 | 608.4 | 361.3 |  | 20.8 | 12.0 | 53.8 | 0.611 | 23.9 | 23.7 | 30.5 |
| F | Chow | 93.6* | 3.7* | 0.284* | 1.32 | 3.05 | 1.31 | 1.99 | 1.31 | 1.96 | 41.5 | 20.1 | 88.4 | 57.1 | 130.9 | 485 | 858.6 | 551.4 | 17.8 | 19.8 | 12.6 | 47.2 | 0.687 | 27.8 | 26.6 | 32.0 |
| F | Chow | 115.5* | 4.4* | 1.727* | 1.32 | 3.78 | 1.39 | 2.02 | 2.01 | 2.09 | 44.7 | 21.5 | 79.0 | 46.9 | 184.6 | 479 | 794.3 | 415.2 | 17.2 | 24.1 | 13.0 | 42.8 | 0.866 | 26.2 | 26.9 | 31.0 |
| F | Chow | 99.1* | 3.9* | 5.279* | 1.42 | 3.45 | 1.45 | 2.19 | 1.78 | 2.01 | 39.3 | 17.7 | 80.9 | 48.6 | 177.6 | 451 | 924.3 | 572.8 | 21.1 | 20.0 | 11.7 | 45.6 | 0.695 | 24.8 | 24.5 | 27.0 |
| F | Chow | 100.5* | 4.3* | 4.819* | 1.33 | 4.58 | 1.39 | 2.04 | 2.52 | 2.12 | 69.5 | 30.6 | 76.2 | 44.8 | 246.3 | 440 | 901.5 | 655.4 | 31.1 | 21.3 | 14.2 | 56.5 | 0.627 | 32.8 | 32.6 | 43.0 |
| F | Chow | 100.3* | 3.5* |  | 1.58 | 3.01 | 1.60 | 2.29 | 1.28 | 2.12 | 33.1 | 15.0 | 88.7 | 57.4 | 176.7 | 455 | 484.6 | 257.2 | 17.0 | 22.8 | 11.7 | 53.3 | 0.646 | 28.20 | 33.2 | 35.0 |
| F | Chow |  |  |  | 1.54 | 2.68 | 1.72 | 2.05 | 1.32 | 2.04 | 25.5 | 11.4 | 83.8 | 50.8 | 160.2 | 449 | 697.5 | 429.3 | 14.4 | 26.3 | 14.0 | 43.8 | 0.921 |  |  |  |
| F | Chow |  |  |  | 1.15 | 3.38 | 1.14 | 1.76 | 1.72 | 1.64 | 35.0 | 15.6 | 81.6 | 49.2 | 120.9 | 447 | 696.3 | 475.7 | 15.0 | 25.3 | 15.8 | 50.8 | 0.809 | 22.0 | 27.0 | 35.0 |

Interventricular septum wall thickness at end-diastole, IVSd; Left ventricular internal diameter at end-diastole, LVIDd; Left ventricular posterior wall thickness at end-diastole, LVPWd;

Interventricular septum wall thickness at end-systole, IVSs; Left ventricular internal diameter at end-systole, LVIDs; Left ventricular posterior wall thickness at end-systole, LVPWs; Ejection fraction, EF; Fractional shortening, FS; Left ventricular, LF; Beat per minute, BPM; Isovolumetric relaxation time, IVRT; Isovolumetric contraction time, IVCT; Myocardial performance index, TEI Index; Systolic velocity at the mitral annulus, s’; *Data not on the same subset of animals as echocardiography.
