# Supplementary figures and images for: Cardiovascular sexual dimorphism in a diet-induced type 2 diabetes rodent model, the Nile rat (Arvicanthis niloticus)
Source: PLoS One. 2018 Dec 27;13(12):e0208987. doi: 10.1371/journal.pone.0208987 (PMC6307866; doi:10.1371/journal.pone.0208987)

(A)


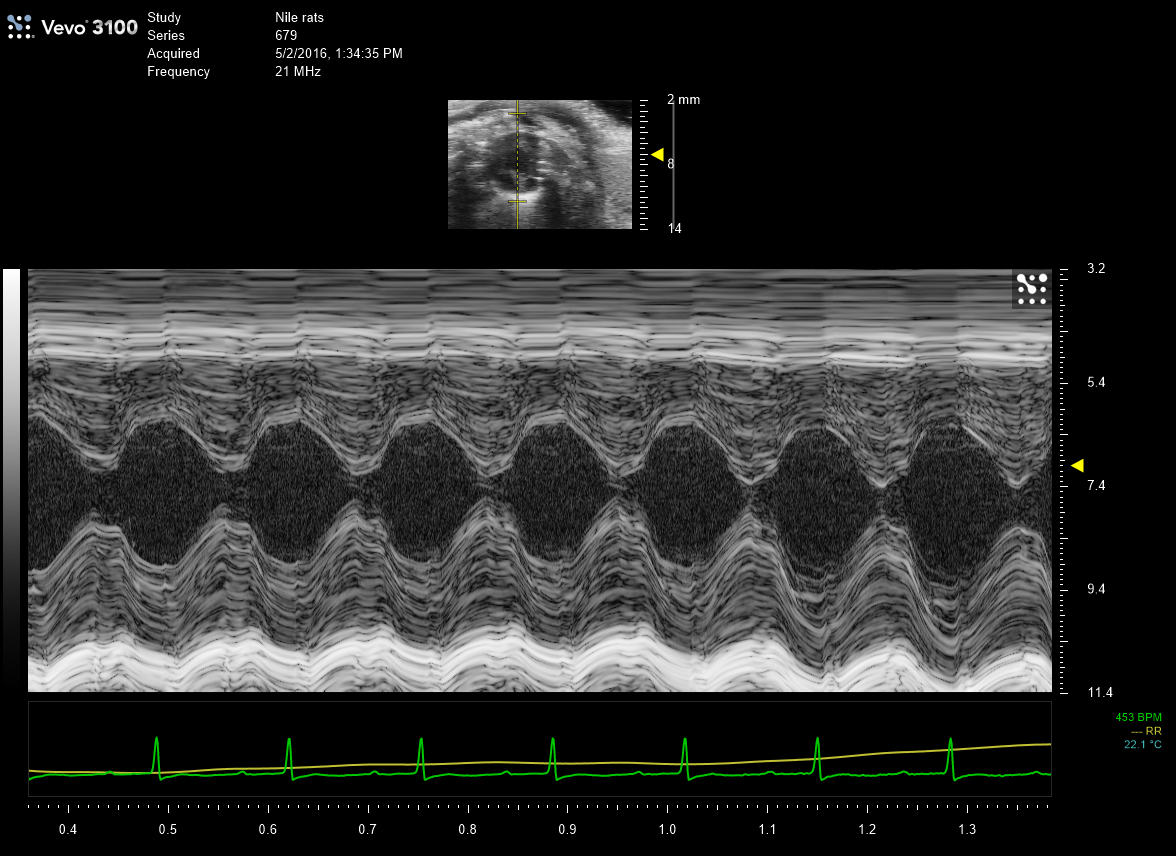


(B)


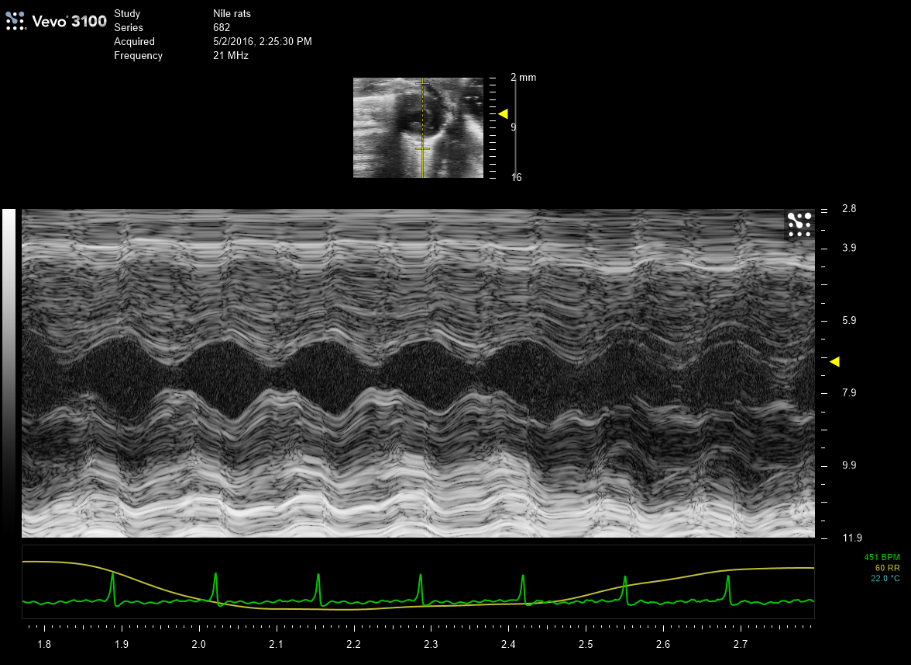


(C)


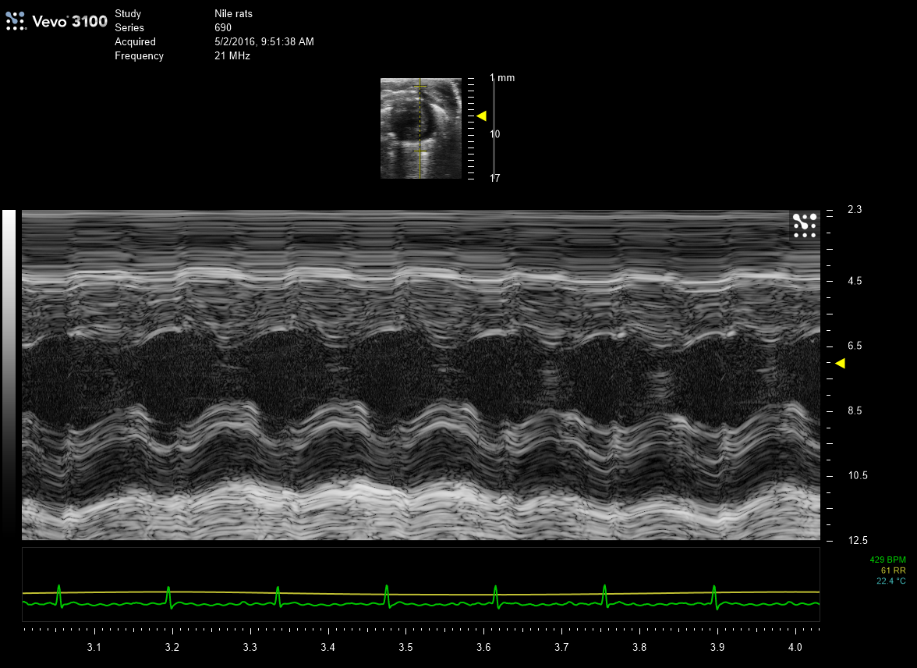


S1 Fig.

Supplement: S1 Fig — The image was from the heart of (A) a female in the Chow group, (B-C) two males in the Chow group. (DOCX) [file pone.0208987.s002.docx]

(A)


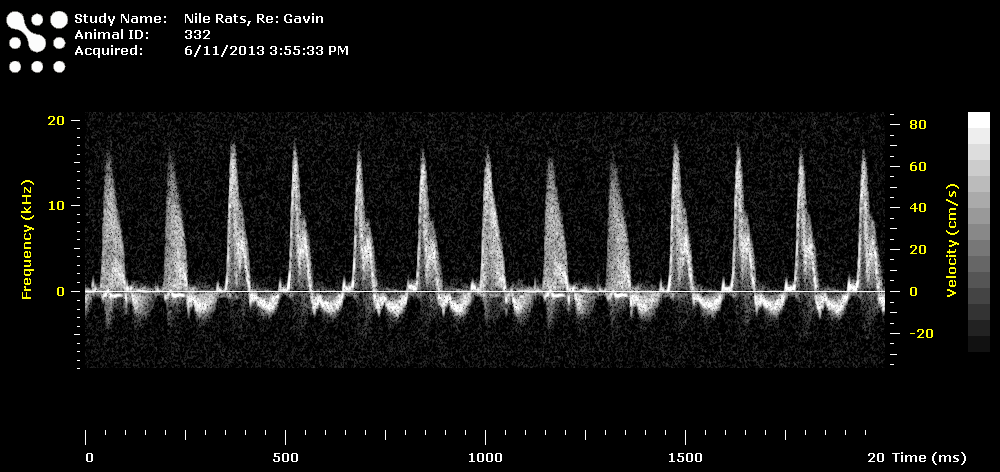


(B)


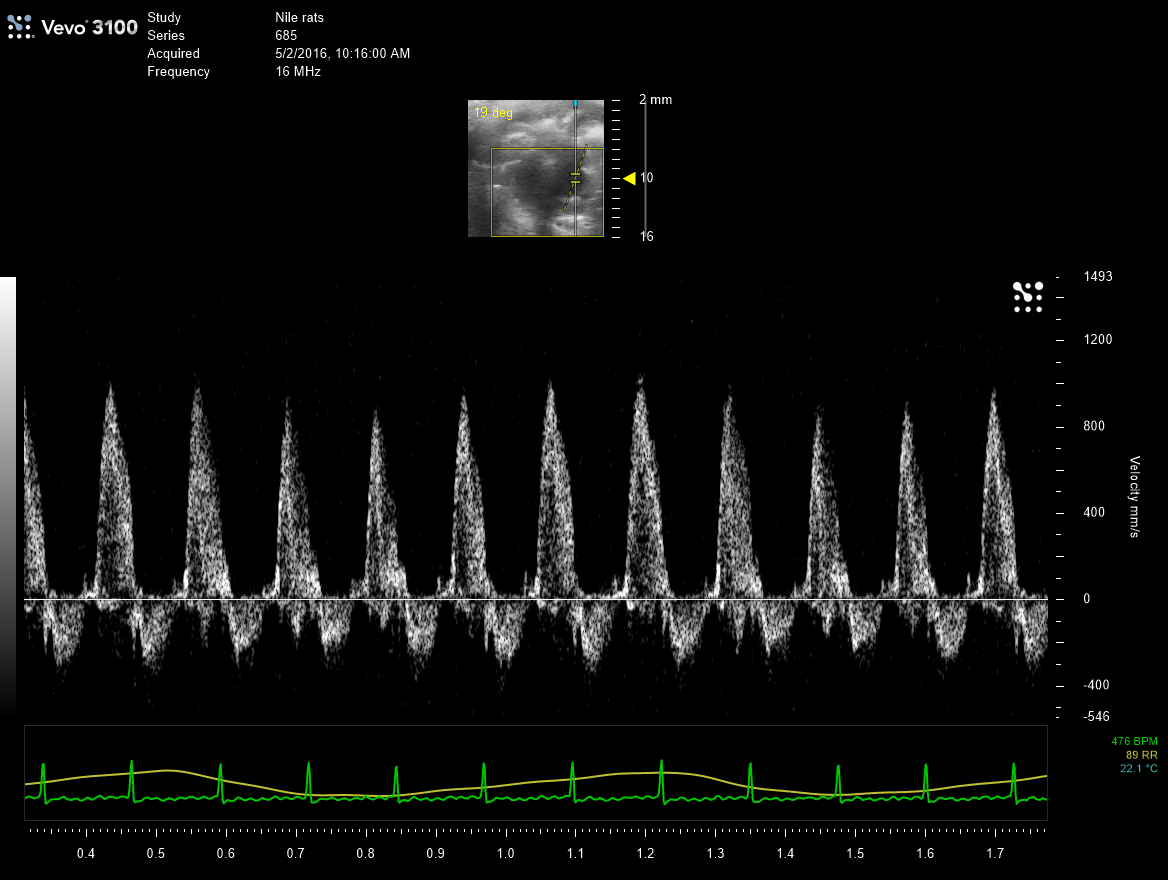


(C)


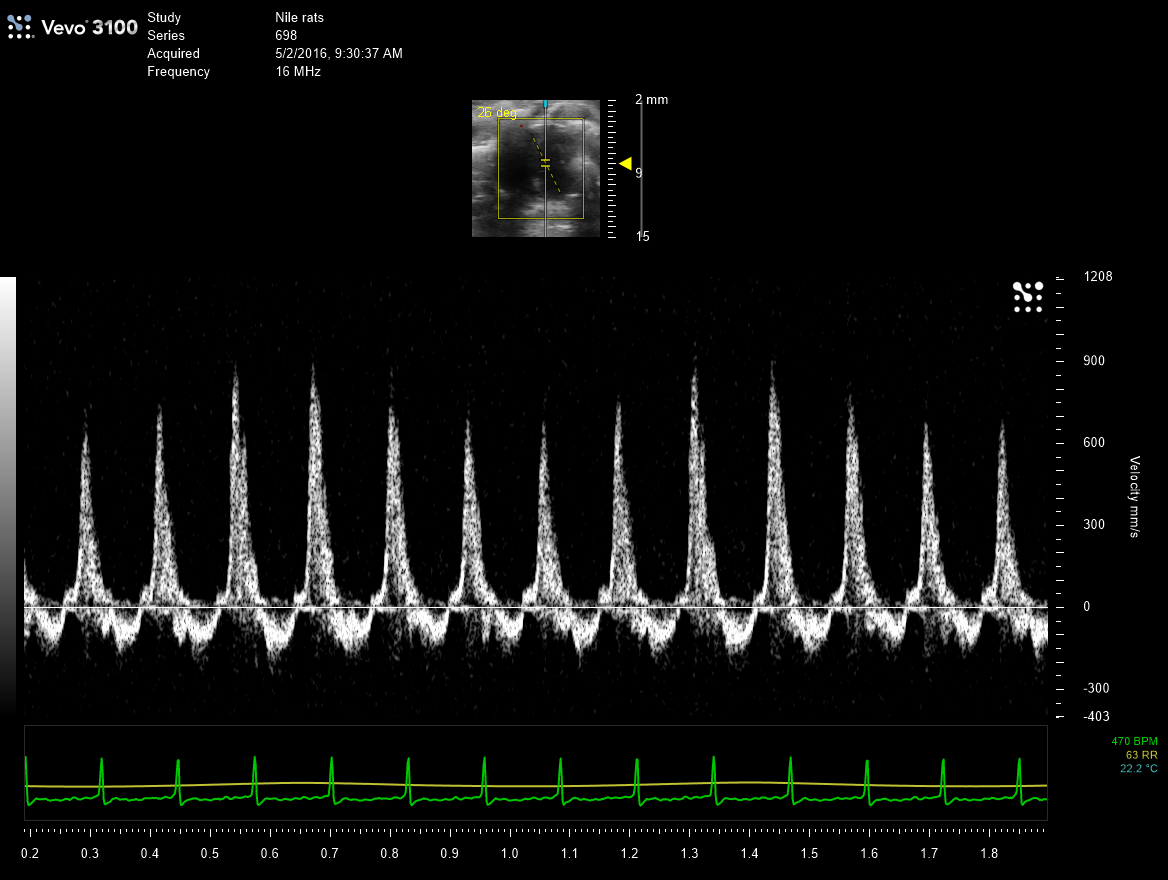


S2 Fig.

Supplement: S2 Fig — The images are from the heart of (A) a female in the Fiber group, (B) a female in the Chow group, and (C) a male in the Chow group. (DOCX) [file pone.0208987.s003.docx]

A)


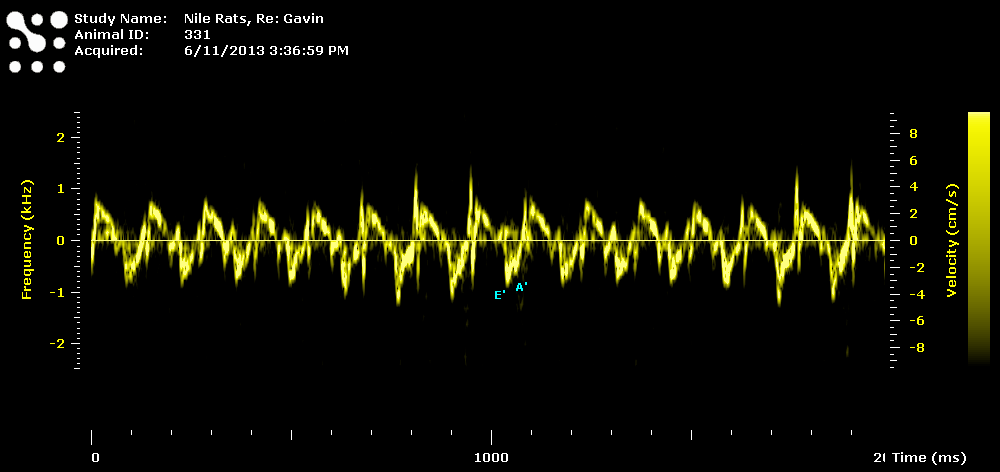


B)


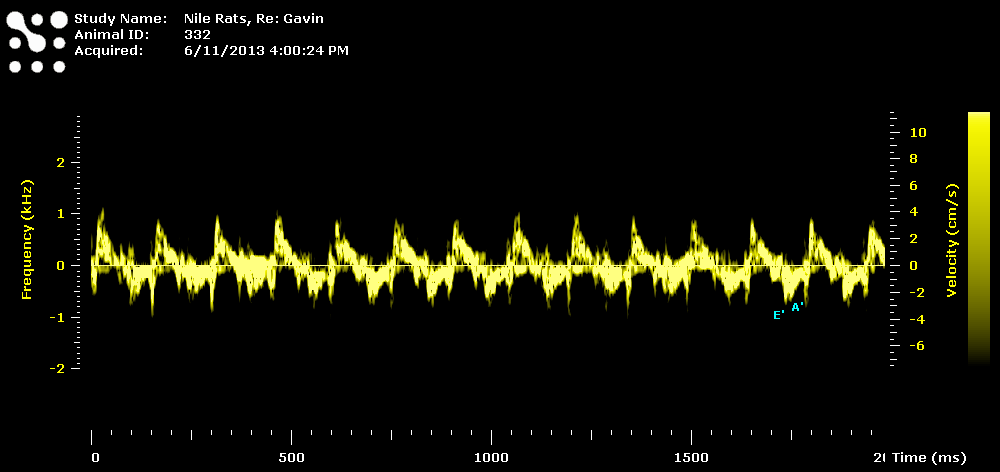


S3 Fig.

.

Supplement: S3 Fig — The images are from the heart of (A) a male in the Fiber group and (B) a female in the Fiber group. (DOCX) [file pone.0208987.s004.docx]
